# Olfactory Proteins Mediating Chemical Communication in the Navel Orangeworm Moth, *Amyelois transitella*

**DOI:** 10.1371/journal.pone.0007235

**Published:** 2009-09-30

**Authors:** Walter S. Leal, Yuko Ishida, Julien Pelletier, Wei Xu, Josep Rayo, Xianzhong Xu, James B. Ames

**Affiliations:** 1 Department of Entomology, University of California Davis, Davis, California, United States of America; 2 Department of Chemistry, University of California Davis, Davis, California, United States of America; UCLA - Physiological Science, United States of America

## Abstract

**Background:**

The navel orangeworm, *Amyelois transitella* Walker (Lepidoptera: Pyralidae), is the most serious insect pest of almonds and pistachios in California for which environmentally friendly alternative methods of control — like pheromone-based approaches — are highly desirable. Some constituents of the sex pheromone are unstable and could be replaced with parapheromones, which may be designed on the basis of molecular interaction of pheromones and pheromone-detecting olfactory proteins.

**Methodology:**

By analyzing extracts from olfactory and non-olfactory tissues, we identified putative olfactory proteins, obtained their N-terminal amino acid sequences by Edman degradation, and used degenerate primers to clone the corresponding cDNAs by SMART RACE. Additionally, we used degenerate primers based on conserved sequences of known proteins to fish out other candidate olfactory genes. We expressed the gene encoding a newly identified pheromone-binding protein, which was analyzed by circular dichroism, fluorescence, and nuclear magnetic resonance, and used in a binding assay to assess affinity to pheromone components.

**Conclusion:**

We have cloned nine cDNAs encoding olfactory proteins from the navel orangeworm, including two pheromone-binding proteins, two general odorant-binding proteins, one chemosensory protein, one glutathione S-transferase, one antennal binding protein X, one sensory neuron membrane protein, and one odorant receptor. Of these, AtraPBP1 is highly enriched in male antennae. Fluorescence, CD and NMR studies suggest a dramatic pH-dependent conformational change, with high affinity to pheromone constituents at neutral pH and no binding at low pH.

## Introduction

Insects are biosensors par excellence. They have developed a remarkable ability to detect with extreme sensitivity and selectivity small, hydrophobic molecules that convey essential information for their reproduction and survival. Female moths, for example, advertize their readiness to mate by releasing infinitesimal amounts of a species-specific sex pheromone bouquet, which is remotely detected by males with remarkable precision. Minute amounts of signal deters eavesdropping, but requires such a fine tuning that the male olfactory system may be considered a “gold standard” in olfaction. It has been estimated that males of the silkworm moths, for example, can detect one molecule of the pheromone bombykol [1]. Moreover, small modifications in pheromone molecules render them completely inactive, or at least a few order of magnitude less active [2]. There is growing evidence in the literature suggesting that pheromone-binding proteins (PBPs) contribute to the sensitivity and possibly the selectivity of the olfactory system. PBPs are part of a family of olfactory proteins, including odorant-binding proteins (OBPs) and chemosensory proteins (CSPs), postulated to be involved in uptake of odorants, transport through the sensillar lymph, and delivery to membrane-bound odorant receptors.

A detailed mechanism has been proposed for a pheromone-binding protein of the silkworm moth, BmorPBP1, suggesting that a pH-dependent conformational change is involved in pheromone binding and release [3], [4], [5], [6]. Indeed, structural biology studies showed that the C-terminal part of the protein forms an additional α-helix at low pH that competes with pheromone molecules for the binding pocket [7], [8], [9], thus enabling the delivery of the pheromone in acidic environment similar to that formed by the negatively charged dendrite surfaces of the olfactory receptor neurons [10]. Functional studies also showed that BmorPBP1, when co-expressed with pheromone receptor BmorOR1 in the empty neuron system of *Drosophila*, enhanced the response to the pheromone, indicating that OBPs contribute to the remarkable sensitivity of the insect's olfactory system [11].

The navel orangeworm, *Amyelois transitella* Walker (Lepidoptera: Pyralidae), is the most serious insect pest of almonds and pistachios in California, and a major pest of a number of other crops, including walnuts and figs. The navel orangeworm is primarily controlled during the growing season with pyrethroids and insect growth regulators, but alternative methods of control are sorely needed. Sex pheromones offer an environmentally-friendly alternative to control insect populations by mating disruption or other strategies in integrated pest management. Typically, sex pheromones and other attractants (aka semiochemicals) are identified by a bioassay-guided isolation of natural products. Alternatively, olfactory proteins may be used in a reverse chemical ecology approach [12], [13] for screening potential attractants on the basis of their affinity to odorant-binding proteins. These proteins are part of a large family of carrier proteins, for which we coined the term encapsulins [12], [14], but those directly involved in semiochemical reception are grouped into pheromone-binding proteins (PBPs) and general odorant-binding proteins (GOBPs) based on their transport of pheromones or other semiochemicals [15], respectively. We have now isolated, cloned and expressed olfactory proteins from the navel orangeworm and set the stage to use them in reverse chemical ecology. Although the sex pheromone system of the navel orangeworm has already been identified [16], [17], some of the constituents are unstable. Reverse chemical ecology in this case can be used for the development of alternative compounds (parapheromones).

## Results and Discussion

### Isolation of antennae-specific proteins

**Figure 1:**
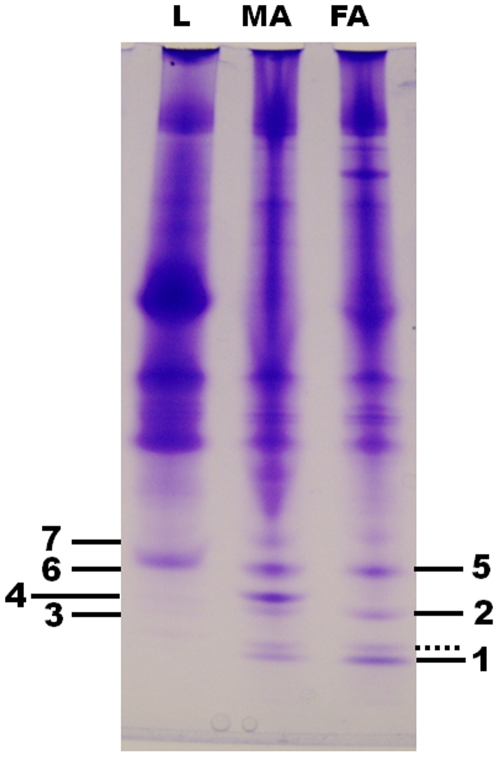
Analysis of proteins extracted from olfactory and non-olfactory tissues. Protein extracts were separated on a 15% native PAGE and stained with Coomassie Brilliant Blue. Seven bands were tentatively identified as antennae-specific. A band marked with a dotted line, with slower migration than Band 1, was ruled out because the faint band in leg extracts was clearly detected in extracts from large samples of this non-olfactory tissue. L, extract from male hindlegs (15 legs-equivalent); MA, male antennae (70 antennae-equivalent); FA, female-antennae (65 antennae-equivalent).

To isolate putative olfactory proteins from the navel orangeworm, we extracted proteins from olfactory and non-olfactory tissues dissected from adult males and females, and compared protein profiles of these extracts by native polyacrylamide gel electrophoresis (PAGE). Typically, OBPs are abundant acidic proteins that migrate faster than non-olfactory proteins thus appearing in the lower part of a native gel. Antennae-specific proteins can be identified by comparing protein extracts from non-olfactory tissues (e.g.: legs) with protein profiles from antennae. In addition, comparison of male and female antennal extracts may identify putative PBPs, which in most cases are specifically expressed or at least enriched in male antennae. This protein-based approach led us to identify several bands (Bands 1–7), which are likely to represent olfactory proteins from the navel orangeworm (Fig. 1). A faint band migrating just above Band 1 (Fig. 1) was also detected in leg extracts when larger control samples were analyzed (data not shown). To obtain the N-terminal amino acid sequences of the target proteins by Edman degradation we re-ran native PAGE analysis, transferred proteins to polyvinyl difluoride membranes and isolated the bands after staining. Band 1 (Fig. 1) was slightly more intense in extracts from female antennae, but samples from male and female antennae gave the same N-terminal sequence: SAEVMSHVTAHFGKA. Contrary to the initial assumption that Bands 2 and 3 were different, they gave the same N-terminal sequence: SQEVLHKMTASF. On the other hand, Band 4 was detected exclusively in male antennae and gave the N-terminal sequence SPEIMKDLSINFGKA. Bands 5 and 6 gave identical N-terminal sequences, DVAVMKDVTLGFGEA with nearly equal intensity in extracts from male and female antennae, whereas Band 7 (SDYKTGKIENINIQE) was detected with higher intensity in male than female antennae.

### Cloning of PBPs, GOBPs and a Chemosensory Protein (CSP)

Our PCR approach to clone the cDNAs encoding the isolated proteins started with the isolation of total RNA from antennal tissues and synthesis of first strand cDNA by the SMART RACE cDNA Amplification. We used 3′-RACE cDNA and 5′-RACE cDNA templates with degenerate primers, designed on the basis of the identified N-terminal amino acid sequences, GCGT_15_ or universal primer mix (UPM) primers and, subsequently, gene-specific primers (GSPs). With primers designed on the basis of the male-specific Band 4 we obtained a 711 bp-long cDNA encoding 164 amino acid residues, including 22 residues of a signal peptide, which was assigned on the basis of the N-terminal sequence of the mature protein. The mature protein contained the hallmark of insect OBPs, six cysteine residues. Blastp search indicated that this protein had 72, 70, and 69% identities with *Synanthedon exitiosa* PBP (AAF06142) [18], *Antheraea polyphemus* PBP1 (CAA35592) [19] and *A*. *pernyi* PBP2 (Q17078) [20]. Thus we named this protein AtraPBP1 (Accession Number, GQ433364; calculated molecular weight, 16,072 Da; pI, 4.99).

Next, we cloned the genes encoding the other antennae-specific proteins starting with Band 1. Although we observed a rather unusual connection of the SMART II Oligonucleotide (Clontech) to the 5′-end of the cDNA immediately after the 5′-region encoding the N-terminal amino acid sequence, we were able to isolate a 511 bp-long cDNA encoding a 141-residue mature protein with a N-terminal sequence identical to that of the isolated protein. The translated protein had six cysteine residues and showed 84, 81 and 79% identities with *Manduca sexta* GOBP2 (AAG50015) [21], *A. pernyi* GOBP2 (Q17075) [22], and *Samia cynthia ricini* GOBP2 (BAF91328) (Leal, unpublished), respectively. We, therefore, named the Band 1 protein AtraGOBP2 (Accession Number, GQ433368; calculated molecular weight, 16,166 Da; pI, 4.87).

We had also encountered problems with 5′-RACE when cloning the cDNA encoding the protein in Bands 2 and 3, but in this case the SMART II Oligonucleotide was connected upstream of the 5′-region encoding the N-terminal amino sequence and the signal peptide. The cloned partial cDNA sequence included 614 bp encoding for 21 amino acid residues of the signal peptide and 146 residues of the mature protein. We sequenced 16 independent clones and observed four points of polymorphism at 138^th^ C/G, 368^th^ T/A, 521^st^ A/T and 539^th^ T/A, which suggest the occurrence of two forms of the mature protein, one with Phe-102 and the other having Tyr-102, and both having six cysteine residues. Blastp search indicated 67, 66, and 65% identity to pheromone-binding proteins from *Heliothis virescens* PBP2 (CAL48346) [23], *Helicoverpa assulta* PBP2 (ABY28381) (Yang, W. L. et al., unpublished), and *H. armigera* PBP2 (ACD01993) (Zhang, S. et al, unpublished), respectively. Therefore, we named the two forms AtraPBP2F102 (Accession Number, GQ433365; calculated molecular weight, 16,731 Da; pI, 5.15) and AtraPBP2Y102 (Accession Number, GQ433366; calculated molecular weight, 16,747 Da; pI, 5.15).

With degenerate primers for the protein in Bands 5 and 6, we isolated a partial cDNA sequence of 702 bp encoding 144 amino acid residues of the mature protein, which included seven cysteine residues, and four residues of the signal peptide. Blastp search indicated that the protein had 78, 77, and 76% identities with GOBP1s from *Bombyx mori* (CAAA64444) [24], *Plutella xylostella* (ABW05104) (Dong, X.-L. et al., unpublished) and *H. virescens* (CAA65605) [25]. These data suggest that the protein in Bands 5 and 6 belongs to the seven-cysteine group of GOBP1s from moths. Consequently, we named this protein AtraGOBP1 (Accession Number, GQ433367; calculated molecular weight, 16,903 Da; pI, 5.11).

Lastly, we cloned the cDNA encoding the protein detected in Band 7. A partial 537 bp-long cDNA was isolated, which encoded for 105 amino acid residues, including the N-terminal sequence obtained by Edman degradation. This protein was named AtraCSP (Accession Number, GQ433369; calculated molecular weight, 12,923 Da; pI, 5.63) because the mature protein contains four cysteine residues and showed 80, 77, and 73% amino acid identity to chemosensory proteins from *B. mori* (AAV34688) [26], *Cactoblastis cactoris* (AAC47827) [27], and *H. armigera* (AF368375) (Deyts et. al., unpublished), respectively.

### Cloning of other olfactory proteins

While attempting to clone the cDNA encoding AtraCSP, we isolated a cDNA fragment encoding a glutathione S-transferase. Because these enzymes are implicated in odorant reception [28], we have designed gene-specific primers, obtained the entire sequence by 5′-RACE, and named this protein AtraGST (Accession Number, GQ433371).

Using a degenerate primer PCR approach, we have identified a partial cDNA sequence encoding a putative sensory neuron membrane protein (SNMP). AtraSNMP1 (942 bp, 314aa) (Accession Number, GQ451327) displays 75% amino-acid identity to *Mamestra brassicae* SNMP1 (AF462066) (Jacquin-Joly, E. et al., unpublished) and *H. virescens* SNMP1 (AJ251959) and 74% to *B. mori* SNMP1 (AJ251958) [29]. Likewise, we have identified a partial cDNA sequence encoding a putative atypical OR83b-like odorant receptor 2 (OR2). AtraOR2 (813 bp, 271aa) (GQ451328) is highly conserved, sharing 85 to 91% amino-acid identity with orthologs of other lepidopteran species. While attempting to clone cDNAs encoding pheromone receptors using degenerate forward primer and universal primer, we have identified a partial cDNA sequence encoding a putative antennal binding protein X (ABPX). AtraABPX (421 bp, 117aa) (Accession Number, GQ451326) displays 69% amino-acid identity to *H. virescens* ABPX (AJ002518) [30], 64% to *Agrotis ipsilon* ABPX (AY301981) (Picimbon, J.-F. et al., unpublished) and 63% to *B. mori* ABPX (X94990) [31].

### Expression patterns of olfactory proteins and phylogenetic relationships

**Figure 2:**
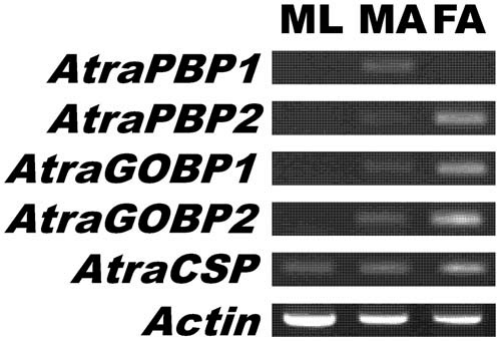
Gene expression analysis by RT-PCR. Expression of *AtraPBP1, AtraPBP2, AtraGOBP1, AtraGOBP2*, and *AtraCSP* genes in control tissue (ML, male hindlegs) and olfactory tissues (MA, male antennae and FA, female antennae). *Actin* gene was used as endogenous control.

**Figure 3:**
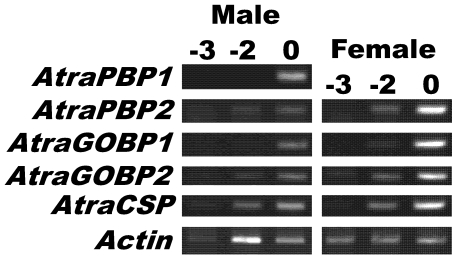
Expression of olfactory genes during late pupal-adult development. RT-PCR analysis of *PBPs*, *GOBPs*, and *CSP* genes in antennae of late pupae (day -3, and day -2) and newly emerged adults (day 0) as indicated on the top of the gels as -3, -2, and 0, respectively. Due to unusually high RNAse activity it was not possible to generate cDNA templates for day -1, and despite considerable efforts there was a fluctuation in cDNA amounts, as indicated by *actin* detection.

To compare transcript patterns with protein profiles (Fig. 1), RT-PCR experiments were performed using gene-specific primers. First, we compared expression of *AtraPBP1, AtraPBP2, AtraGOBP1, AtraGOBP2*, and *AtraCSP* in non-olfactory tissues (male legs) with olfactory tissues (male and female antennae) (Fig. 2). In general, gene expression mirrored protein profiles, except for *AtraCSP*, which was detected not only in male and female antennae, but also in non-olfactory tissues (legs). *AtraPBP2, AtraGOBP1* and *AtraGOBP2* genes were detected in both male and female antennae, but not in legs, whereas *AtraPBP1* was apparently expressed exclusively in male antennae. Next, we assessed gene expression during antennal development. Contrary to our previous experience with the wild silkworm moth, *A. polyphemus* [32], sampling antennal pockets from pupae and day 0 adults of the navel orangeworm and extracting RNA were very challenging due to high RNAse activity at this developmental stage as reflected in the irregular amplifications of *actin* control gene (Fig. 3). Indeed, we were unable to extract RNA sample just the day before adult eclosion (day -1). Despite the unavoidable fluctuation in template titers, these experiments suggest that gene expression of most olfactory proteins starts at least two days before adult emergence (Fig. 3). Expression of the male antennae-specific *AtraPBP1* starts at day 0 of adult stage or the day prior to adult emergence.

**Figure 4:**
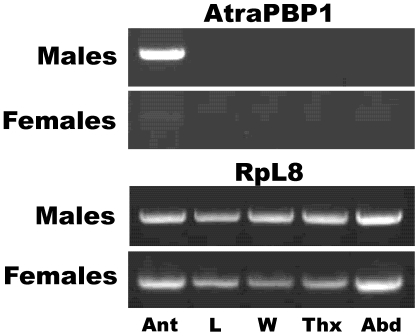
RT-PCR analysis of a pheromone-binding protein gene. As previous results suggested that *AtraPBP1* is expressed exclusively in male antennae, a more stringent RT-PCR analysis was performed and expression in other non-olfactory tissues was re-examined. *AtraPBP1* was detected in male antennae (Ant), but not in legs (L), wings (W), thorax (Thx), or abdomen (Abd). A faint band, hardly seen in the figure, was observed in the original gel with female cDNA template thus suggesting basal expression in female antennae. RpL8 was used as a control gene.

Having observed by non-quantitative RT-PCR that *AtraPBP1* gene is expressed only in male antennae, a more thorough examination of gene expression was performed. Indeed, *AtraPBP1* was limited to expression in male antennae (Fig. 4), with no trace detected in non-olfactory tissues, including legs, wings, thorax, and abdomen. It is worth mentioning, however, that a faint band was observed when cDNA from female antennae was used as template thus suggesting that *AtraPBP1* is highly enriched in male antennae. Consequently, it is reasonable to assume that AtraPBP1 plays male-specific role(s), such as the detection of sex pheromones.

**Figure 5:**
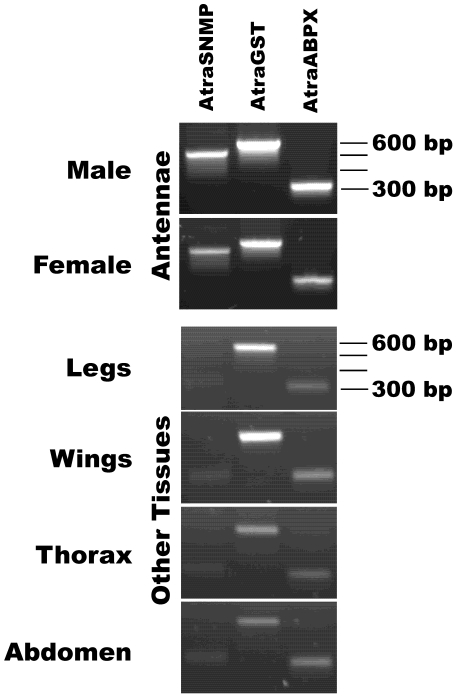
Expression of other olfactory genes in antennal and non-olfactory tissues. RT-PCR shows that *AtraSNMP* (expected PCR fragment, 515 bp) is expressed only in male and female antennae, whereas *AtraGST* (580 bp), *and AtraABPX* (310 bp) genes are expressed not only in male and female antennae, but also with lower intensity in non-olfactory male tissues: legs, wings, thorax, and abdomen. Analysis of *AtraSNMP* expression in other tissues showed a non-specific faint band (ca. 350 bp), which does not correspond in size to SNMP band. Migration of the 300, 400, 500, and 600 bp markers are indicated at the right side gels. Template control is shown if Fig. 4.

**Figure 6:**
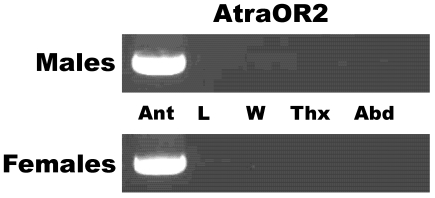
Analysis of *AtraOR2* gene expression by RT-PCR. This co-receptor gene was detected in male and female antennae (Ant), but not in legs (L), wings (W), thorax (Thx), or abdomen (Abd). Template control is shown in Fig. 4.

Next, we assessed tissue-specificity of other olfactory proteins we have isolated by cloning, namely, AtraSNMP1, AtraGST, and AtraABPX. RT-PCR data (Fig. 5) suggest that the genes encoding these proteins are highly expressed in male and female antennae. However, AtraGST and AtraABPX have also been detected, albeit with lower intensity, in all non-olfactory tissues tested (Fig. 5). By contrast, the gene encoding the co-receptor *AtraOR2* was expressed only in male and female antennae, with no trace being detected in non-olfactory tissues (Fig. 6).

**Figure 7:**
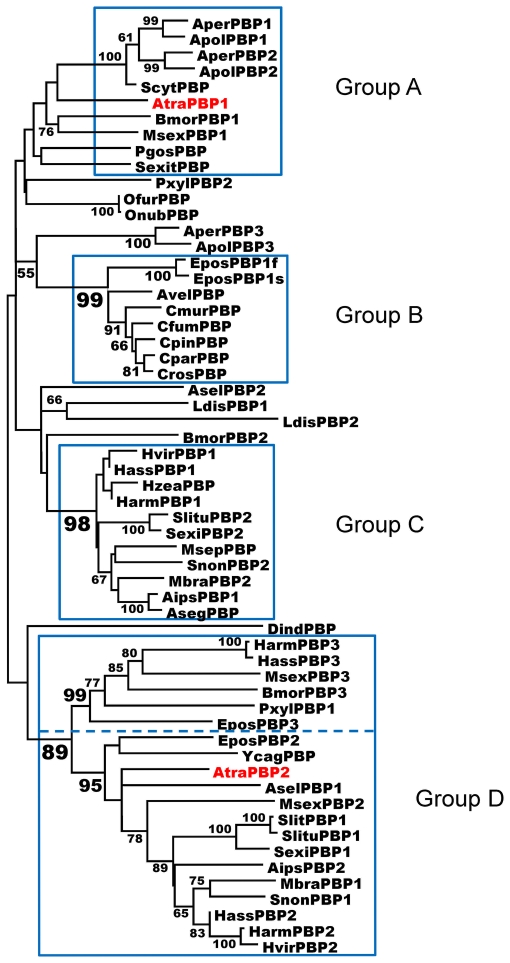
Phylogenetic relationships of moth PBPs. Four groups were identified (A–D). The dashed line in Group D suggests a possible subdivision into D1 and D2. The following PBPs have been included in phylogenetic analysis: *Agrotis ipsilon*: AipsPBP1 (AY301985), AipsPBP2 (AY301986); *Antheraea pernyi*: AperPBP1 (X96773), AperPBP2 (X96860), AperPBP3 (AJ277265); *Antheraea polyphemus*: ApolPBP1 (X17559), ApolPBP2 (AJ277266), ApolPBP3 (AJ277267); *Agrotis segetum*: AsegPBP (AF134292); *Ascotis selenaria*: AselPBP1 (AB285328), AselPBP2 (AB327273); *Argyrotaenia velutinana*: AvelPBP (AF177641); *Bombyx mori*: BmorPBP1 (NM_001044029), BmorPBP2 (AM403100), BmorPBP3 (NM_001083626); *Choristoneura fumiferana*: CfumPBP (AF177642); *Choristoneura murinana*: CmurPBP (AF177646); *Choristoneura parallela*: CparPBP (AF177649); *Choristoneura pinus*: CpinPBP (AF177653); *Choristoneura rosaceana*: CrosPBP (AF177652); *Diaphania indica*: DindPBP (AB263115); *Epiphyas postvittana*: EposPBP1f (AF416587), EposPBP1s (AF416588), EposPBP2 (AF411459), EposPBP3 (EV811597); *Helicoverpa armigera*: HarmPBP1 (AJ278992), HarmPBP2 (EU647241), HarmPBP3 (AF527054); *Helicoverpa assulta*: HassPBP1 (AY864775), HassPBP2 (EU316186), HassPBP3 (DQ286414); *Heliothis virescens*: HvirPBP1 (X96861), HvirPBP2 (AM403491); *Heliothis zea*: HzeaPBP (AF090191); *Lymantria dispar*: LdisPBP1 (AF007867), LdisPBP2 (AF007868); *Mamestra brassicae*: MbraPBP1 (AF051143), MbraPBP2 (AF051142); *Mythimna separata*: MsepPBP (AB263112); *Manduca sexta*: MsexPBP1 (AF117593), MsexPBP2 (AF117589), MsexPBP3 (AF117581); *Ostrinia furnacalis*: OfurPBP (AF133630); *Ostrinia nubilalis*: OnubPBP (AF133637); *Pectinophora gossypiella*: PgosPBP (AF177656); *Plutella xylostella*: PxylPBP1 (FJ201994), PxylPBP2 (AB263118); *Samia cynthia ricini*: ScytPBP (AB039793); *Synanthodon exitiosa*: SexitPBP (AF177657); *Spodoptera littoralis*: SlitPBP1 (EF396284); *Spodoptera litura*: SlituPBP1 (DQ004497), SlituPBP2 (DQ114219); *Sesamia nonagrioides*: SnonPBP1 (AY485219), SnonPBP2 (AY485220); *Spodoptera exigua*: SexiPBP1 (AY743351), SexiPBP2 (AY545636); *Yponomeuta cagnagellus*: YcagPBP (AF177661).

In order to gain insight of the relationships among moth PBPs, we have carried out a phylogenetic analysis in Mega v4.0.2 [33], combining amino acid sequences of the two PBPs from the navel orangeworm (this study) with 57 PBPs previously identified in 33 moth species. A consensus sequence comparison tree was constructed by the neighbor joining method [34] with one thousand bootstrap replicates. The resulting tree suggests that based on their amino acid identity, moth PBPs are clustered into different groups, each comprising related proteins of different moth species (Fig. 7). Indeed, phylogenetic analysis shows the existence of at least four distinct groups of PBPs in moths, illustrating the diversity of this multigenic family. AtraPBP1 and AtraPBP2 belong to two separated groups, with the protein enriched in male antennae, AtraPBP1, clustering with some of the most well-characterized insect PBPs like BmorPBP1 [3], [4], [6], [7], [8], [9], [35], [36] and ApolPBP1 [37], [38], [39], [40]. Despite little boostrap support in the tree, these moth PBPs share 65–70% amino acid identity with AtraPBP1, whereas AtraPBP2 is only 48% identical to AtraPBP1. Contrarily to AtraPBP1, AtraPBP2 belongs to a well supported group (95% bootstrap support) comprising 13 PBPs of other moth species.

### pH-Dependent conformational change and pheromone binding

**Figure 8:**
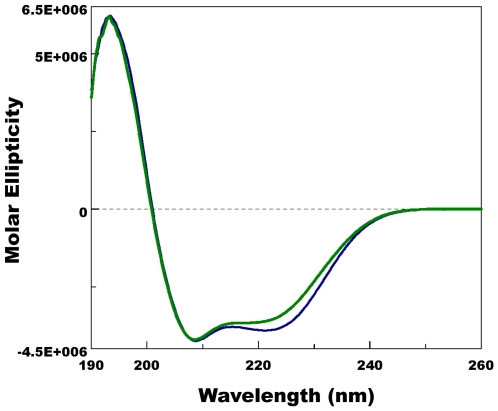
Far-UV CD spectra of AtraPBP1 at pH 7 (blue trace) and pH 5 (green trace). Decrease of a minimum at 223 nm at low pH suggests that this α-helical-rich protein undergoes a pH-mediated conformational change.

**Figure 9:**
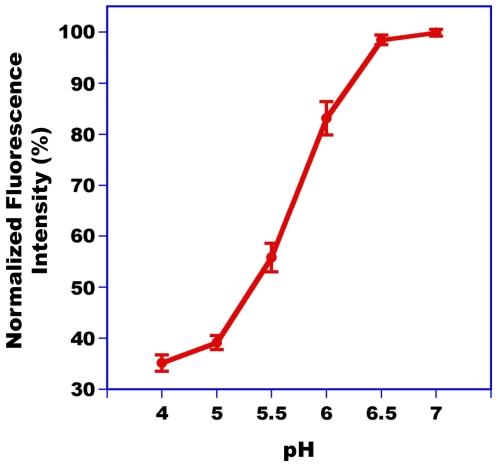
pH-Titration of AtraPBP1 by fluorescence. Intensity of an intrinsic fluorescence emission at 336 nm decreased from high to low pH, with a dramatic transition between 5 and 6.5.

Having previously observed that PBPs from the silkworm moth, *B. mori*, and the wild silkworm moth, *A. polyphemus*, undergo pH-dependent conformational changes [3], [4], [5], [36] that lead to lack of binding at low pH [3], [5], we assessed the effect of pH on the conformation of AtraPBP1. We prepared samples of recombinant AtraPBP1 by using a recombinant pET vector without His6-Tag that generates PBPs with identical conformation and disulfide bridge formation [3] as the native protein. Samples were highly purified by a combination of ion-exchange chromatography (DEAE), high-resolution ion-exchange chromatography (Mono Q), and gel filtration, with the purity confirmed by SDS-PAGE and LC-ESI/MS (>99.5%). We prepared samples for circular dichroism (CD) and fluorescence analysis by taking aliquots of the same sample and diluting with buffers of the desired pH. Far-UV-CD spectrum of AtraPBP1 (Fig. 8) at pH 7 with a maximum at 193 nm and two minima at 208 and 223 nm demonstrated that this PBP is α-helical rich like BmorPBP1 [3] and ApolPBP1 [38]. At lower pH, the intensity of the second minimum at 223 nm was clearly reduced and thus indicated that there is unwinding of helical secondary structure. Similar changes have been observed with CD spectra of BmorPBP1 [3] and ApolPBP1 [38]. Apparently, the formation of a C-terminal helix does not offset the unwinding of the N-terminal α-helix thus causing a reduction in the overall content of this secondary structure. pH-Titration by intrinsic fluorescence (Fig. 9) showed a dramatic transition between pH values of 5 and 6.5 thus suggesting that AtraPBP1 exists in two distinct conformations, one at the pH of the sensillar lymph and the other at low pH as in the vicinity of dendritic membranes [3], [12], [14], [41], [42].

**Figure 10:**
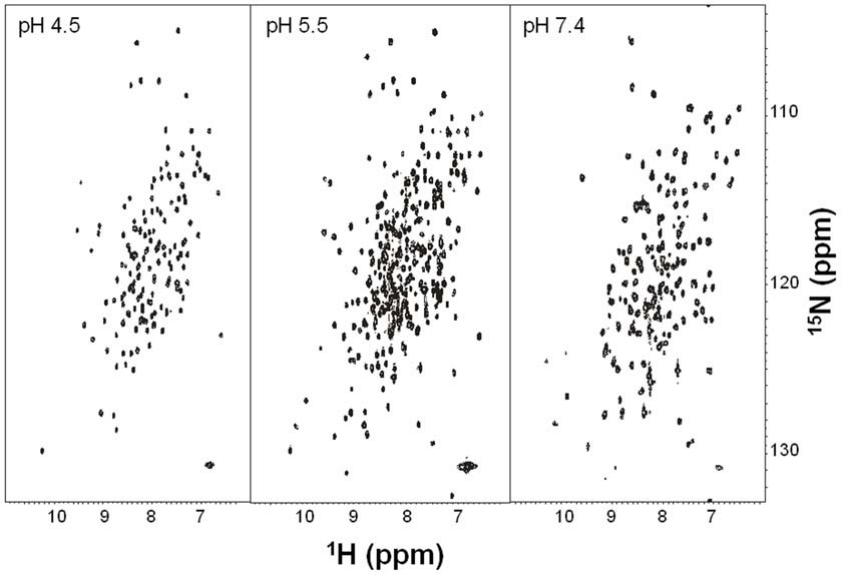
2D-NMR data recorded at high, low, and intermediate pH. A uniform, stable, and monomeric tertiary structure is inferred by the 142 sharp, well-resolved peaks of main-chain amide resonances. The broader peaks at pH 7.4 suggest that AtraPBP1 exists as a heterogenous mixture of monomer, dimer, and multimer, as previously suggested for BmorPBP1 [36]. The NMR spectrum at intermediate pH is in agreement with a transition state with at least two conformations in equilibrium.

NMR analysis revealed very striking spectral changes upon changing the pH from 4.5 to 7.4. The ^15^N-^1^H heteronuclear single quantum coherence spectrum at pH 4.5 (Fig. 10) exhibited the expected number of sharp and well-resolved main-chain amide resonances (142 peaks), indicating the protein forms a uniform, stable, and monomeric tertiary structure at low pH. At pH 5.5, the number of NMR peaks increased almost two-fold (284 peaks), indicative of an equal mixture of protonated and deprotonated forms of the protein at this intermediate pH. The NMR resonances at pH 7.4 appear broadened with chemical shift heterogeneity (185 peaks), suggesting a heterogeneous mixture of protein structures at neutral or slightly acidic pH. Such heterogeneity may be stabilized with a ligand. We are, therefore, pursuing the three-dimensional structures of AtraPBP1 at low and neutral pH by NMR and X-ray crystallography, respectively. We have already determined NMR backbone assignments for AtraPBP1 at low pH [43] and a full structure determination is currently underway. On the other hand, we were able to co-crystallize AtraPBP1 with pheromone constituents and obtain crystals that diffract to atomic resolution thus allowing determination of structures of AtraPBP1-pheromone complexes.

**Figure 11:**
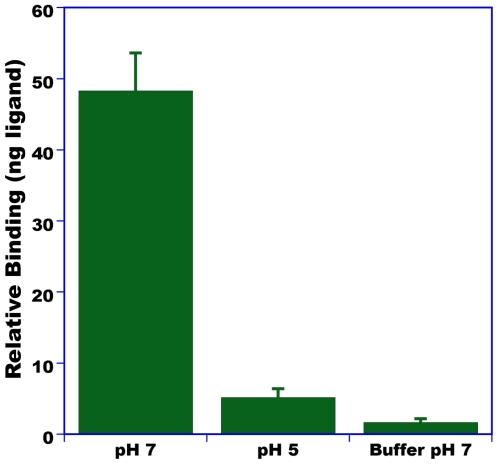
Binding of the major sex pheromone constituent to AtraPBP1. Z11Z13-16Ald showed high affinity for the pheromone-binding protein at pH 7, but low or no binding activity at low pH. Minimal non-specific binding is indicated by the low amounts of ligand detected after incubation with buffer only.

To assess affinity of AtraPBP1 for pheromone constituents, we used a previously developed binding assay [5], which is based on the separation of bound and unbound ligand by a centrifugal device. After the free ligand is removed by filtration, the PBP-bound ligand is released from the protein by lowering the pH, extracted with organic solvent and analyzed by gas chromatography (GC) for quantification and gas chromatography-mass spectrometry (GC-MS) for identification of the bound ligand. The major constituent of the sex pheromone system, (*Z,Z*)-11,13-hexadecadienal, hereafter referred to as Z11Z13-16Ald [16], [17], bound to AtraPBP1 with apparent high affinity at neutral pH (Fig. 11) and low or no binding affinity at low pH. This pH-dependent binding affinity may be explained by the formation of a C-terminal α-helix, which competes with the ligand for the binding cavity at low pH [7], [8], [37]. Although only one of the four isomers of 11,13-hexadecadienal is known to be behaviorally active [17], pheromone-detecting sensilla in male antennae are sensitive to the four isomers of this compound, namely, Z11Z13-16Ald, Z11E13-16Ald, E11E13-16Ald, and E11Z13-16Ald [17]. We compared binding of Z11Z13-16Ald and E11E13-16Ald and found no difference (data not shown) thus suggesting that AtraPBP1 alone cannot discriminate stereoisomers of the major constituent of the sex pheromone. It is not known how many odorant receptors are expressed in the pheromone-detecting sensilla of the navel orangeworm male antennae and if they can discriminate isomers of the major constituent alone or in combination with AtraPBP1.

**Figure 12:**
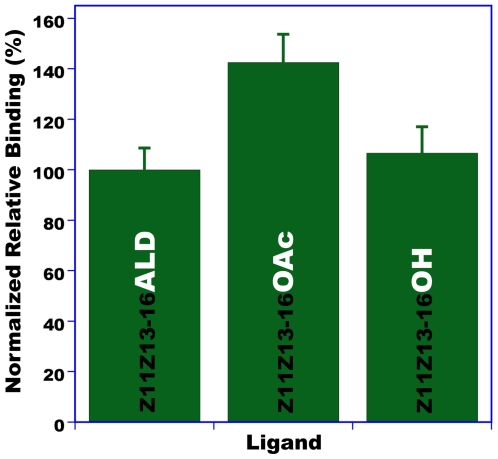
Competitive binding assays with AtraPBP1. Two major constituents of the sex pheromone of the navel orangeworm, Z11Z13-16Ald and Z11Z13-16OH, and a behavioral antagonist, Z11Z13-16OAc, were incubated with AtraPBP1 at the same concentration. The two pheromone constituents bound to AtraPBP1 with nearly, equally high affinity, whereas the behavioral antagonist showed even higher apparent affinity.

Next, we tested binding affinity of other constituents of the navel orangeworm sex pheromone. Female-produced sex pheromones in moths are normally complex mixtures of straight chain acetates, alcohols and aldehydes, with 10–18 carbon atoms and up to three unsaturations, the so-called Type I pheromones. Type II sex pheromone is comprised of polyunsaturated hydrocarbons and epoxy derivatives with long straight chains. The navel orangeworm is unusual in that its sex pheromone system in composed of a complex mixture that includes constituents of both types: Z11Z13-16Ald, Z11Z13-16OH, Z11Z13-16OAc (behavioral antagonist), (*Z*,*Z*,*Z*,*Z*,*Z*)-3,6,9,12,15-tricosapentaene and (*Z*,*Z*,*Z*,*Z*,*Z*)-3,6,9,12,15-pentacosapentaene, and other minor constituents [17]. As opposed to Type I pheromones that gave very low background indicating negligible non-specific binding (see buffer in Fig. 12), it was difficult to assess binding of the pentaene compounds because their hydrophobicity led to high background levels. On the other hand, the secondary constituent, Z11Z13-16OH bound to AtraPBP1 with affinity comparable to that of the major constituent, but showed no affinity at low pH (data not shown). Interestingly, the behavioral antagonist, Z11Z13-16OAc showed the highest affinity to AtraPBP1 of all tested ligands (data not shown). Next, we performed competitive binding studies with AtraPBP1 incubated with the three ligands at the same concentration. These competitive binding assays mirrored what was observed with non-competitive binding assays, AtraPBP1 was bound with the highest affinity to Z11Z13-16OAc, whereas the aldehyde and alcohol showed similar affinity (Fig. 12). These results suggest that a single PBP may be involved in the reception of multiple constituents of sex pheromones.

**Figure 13:**
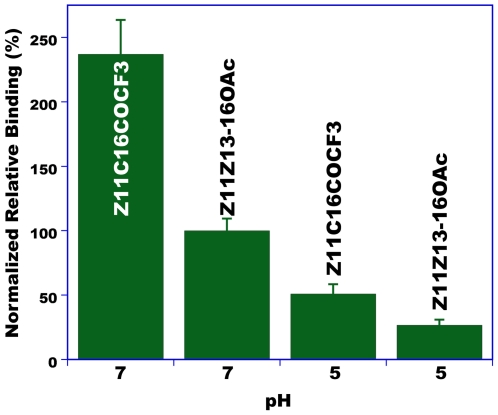
Binding of a pheromone analog to AtraPBP1. Competitive binding of a pheromone analog, Z11C16COCF3, and the best natural ligand, Z11Z13-16OAc. Both ligands at equimolar concentration were incubated with AtraPBP1 at the same time. AtraPBP1 bound with much higher affinity to the pheromone analog and even retained the ligand at low pH.

To further explore the potential use of AtraPBP1 for the development of parapheromones, we tested binding of a pheromone analog, (*Z*)-1,1,1-trifluoro-13-octadecen-2-one (hereafter referred to as Z11C16COCF3). Trifluoromethyl ketones (TFMK) [44] are compounds which inhibit a variety of hydrolytic enzymes, such as acetylcholinesterase, chymotrypsin, trypsin, juvenile hormone esterase, human liver microsomal CEs, and pheromone degrading esterases in male olfactory tissues. They have been demonstrated to interrupt insect chemical communication [45], [46] and to bind to pheromone-binding proteins [47], but their mode of action is still a matter of debate. We compared by competitive binding the affinity of Z11C16COCF3 and Z11Z13-16OAc to AtraPBP1. Surprisingly, Z11C16COCF3 binds to AtraPBP1 with much higher affinity than the behavioural antagonist Z11Z13-16OAc (Fig. 13). Although binding activity decreased dramatically at low pH, this TFMK showed binding affinity at low pH almost half of that of the best natural ligand (Z11Z13-16OAc) at neutral pH (Fig. 13). We, therefore, concluded that AtraPBP1 may be employed for the development of a affinity-based approach for the development of parapheromones.

### Conclusion

We have isolated and cloned olfactory proteins from the navel orangeworm, including pheromone-binding proteins, general odorant-binding proteins, chemosensory protein, antennal binding protein X, glutathione S-transferase, sensory neuron membrane protein and an odorant receptor. Our goal was to identify olfactory proteins involved in the reception of pheromones for future applications in a reverse chemical ecology approach to explore the development of alternative attractants (parapheromones) as substitutes for unstable constituents of the navel orangeworm sex pheromone system. One of the identified olfactory proteins, AtraPBP1, was expressed almost exclusively in male antennae. The major constituent of the sex pheromone, Z11Z13-16Ald bound AtraPBP1 with high affinity at the sensillar lymph pH, but no affinity at the postulated pH at the close vicinity of the pheromone receptor. Because unsaturated aldehydes in general have limited lifetime under UV light and other field conditions, more chemically stable attractants (parapheromones) are needed. AtraPBP1 seems an ideal molecular target for screening parapheromones. Indeed, binding of a pheromone analog, Z11C16COCF3, to AtraPBP1 highlights the potential use of this protein for screening non-natural ligands. The current project paved the way for future structural biology studies aimed at unveiling molecular interactions between AtraPBP1 and Z11Z13-16Ald, and mechanisms of binding and release to set the stage for design of parapheromones.

## Materials and Methods

### Protein identification and characterization

A laboratory colony of the navel orangeworm was initiated from larvae collected in Bakersfield, CA, according to a previously published protocol [48]. Tissues were collected with clean forceps under a microscope, immediately homogenized in 10 mM Tris-HCl, pH 8, with an ice cold Dounce tissue grinder (Wheaton, Millville, NJ) and centrifuged twice at 12,000 xg for 10 min. Samples per batch were typically 50–150 antennae and 50–100 legs. Prior to tissue extraction, adults were sexed [49]. An aliquot of supernatant was concentrated to the appropriate volume with vacuum concentrator and analyzed by 15% native-PAGE. After separations, gels were either stained with Coomassie Brilliant Blue R-250 (CBB, Bio-Rad, Hercules, CA) or proteins were transferred by electroblotting to polyvinyl difluoride (PVDF) membranes, visualized with CBB, bands were cut off, and N-terminal amino acid sequences were obtained on a Precise Protein Sequencing System (Applied Biosystems, Foster City, CA).

### cDNA cloning

Tissues were collected with clean forceps and immediately extracted with TRIzol (Invitrogen, Carlsbad, CA) on an ice-cold Dounce tissue grinder. First strand DNA was synthesized from total RNA using reverse transcriptase and a SMART™ RACE cDNA Amplification Kit (Clontech, Mountain View, CA). 3′-RACE PCR was carried out with appropriate template and degenerate primers based on N-terminal amino acid sequence of the target cDNA, UPM primer, or GCGT_15_ primer. Taq DNA polymerase (ID Labs, London, ON, Canada), *PfuUltra* HotStart DNA polymerase (Stratagene, Cedar creek, TX) and Advantage GC-2 Polymerase Mix (Clontech) were used as polymerases for PCR. The PCR products were subcloned into pBluescript SK(+) (Stratagene) and sequenced. 5′-RACE PCR was performed according to instruction manual using gene specific primers designed on the basis of the sequences obtained by 3′-RACE. Multiple (10–16) independent clones were sequenced to eliminate possible PCR-derived mutations. For cloning AtraPBP1, one degenerate primer and two gene-specific primers were designed: 5′-GA(A/G)AT(A/C/T)ATGAA(A/G)GA(C/T)TT(A/G)TC(A/C/G/T)AT(A/C/T)AA(C/T)TT(C/T)GG -3′ (based on EIMKDLSINFG); AtraPBP1-1, 5′-CTCACAGGCTGTGCCATCAAGTGTCTCTC-3′; AtraPBP1-2, 5′-CAACTTCCATGTTAGGAGCCCATTTGAGG-3′. For AtraPBP2, the following primers were used:

5′-CA(A/G)GA(A/G)GT(A/C/G/T)TT(A/G)CA(C/T)AA(A/G)ATGAC(A/C/G/T)GC-3′ (based on QEVLHKMTA); AtraPBP2-1, 5′-ATCATGTGCATGGCCGCCAAGCTGGACCTG-3′; AtraPBP2-2, 5′-CCACGTCCAGGGTGCGGGCGCAGTGGTCGC-3′; AtraPBP2-4, 5′-TCTGATGTTACAAATATCACGATCAAATCC-3′; AtraPBP2-6, 5′-GCGTTAAGATGGCCACTTGTCGTGTGCGTG-3′

For AtraGOBP1: degenerate primer,5′-AA(A/G)GA(C/T)GT(A/C/G/T)AC(A/C/G/T)CT(A/C/G/T)GG(A/C/G/T)TT(C/T)GG(A/C/G/T)GA(A/G)GC-3′ (based on KDVTLGFGEA), AtraGOBP1-1, 5′-AAAAGTGACCGTTGCATAGAAGCTTATGCG-3′.

For AtraGOBP2, 5′-CA(C/T)GT(A/C/G/T)AC(A/C/G/T)GC(A/C/G/T)CA(C/T)TT(C/T)GG(A/C/G/T)AA(A/G)GC-3′ (based on HVTAHFGKA); AtraGOBP2-1, 5′-GAAGTGGTGGACCGCGAGCTGGGCTGCGCC-3′; AtraGOBP2-2, 5′-CATTTGCACCATTCTCTGGGAGAGGACC-3′.

For AtraCSP two degenerate primers were used, the first being 5′-GT(A/C/G/T)AA(A/G)AT(A/C/T)GA(A/G)AA(C/T)AT(A/C/T)AA(C/T)AT(A/C/T)CA(A/G)GA-3′ (based on GKIENINIQE) generated a 1-kb-long PCR fragment by misannealing that led to cloning AtraGST. A second degenerate primer was then designed: 5′-GA(C/T)TA(C/T)AA(A/G)AC(A/C/G/T)GG(A/C/G/T)AA(A/G)AT(A/C/T)GA(A/G)AA(C/T)AT(A/C/T)AA(C/T)AT(A/C/T)CA(A/G)GA(A/G)-3′ (based on DYKTGKIENINIQE). The following GSP were also employed: AtraGST1-1, 5′-GGGCAAGCAGTGGTAACAACGCAGAGTAG-3′; AtraGST1-2, 5′-CTCTATCTCACGAAAAAGTAAAGAG-3′, AtraCSP1-1 5′-GGGCAAGTGCACGCCGGAAGGAAAGGAAC-3′. For AtraSNMP1, the following forward and reverse degenerate primers were used: AtraSNMP1-1 5′-GA(A/G/C/T)GAATGGAAAGA(A/G)AAGGT(A/G)GA-3′; AtraSNMP1-2 5′-AGCAT(C/G/T)TTCAC(A/G)AA(C/G/T)GT(C/T)TTGTT-3′, respectively. Likewise,

AtraOR2-1: 5′-ACCCT(C/G)GCAGT(A/G)TGGAA(C/T)CAGTC-3′ and

AtraOR2-2: 5′-CTGGCA(C/T)TGTTGGCA(C/G)ACGATCTG-3′ were used for AtraOR2 and AtraPR1-1: 5′- T(A/G)CC(A/G)TGGGA(A/G)(A/G/T)(A/C/G))(C/T)ATGGA-3′ for AtraABPX cloning.

### RT-PCR

cDNAs were prepared from freshly extracted tissues from male legs, male antennae, and female antennae. For developmental studies, two antennae or antennal pockets with antennae and one hindleg were collected from day -3, day -2, and day 0 adult. It was not possible to extract RNA from day -1 adult because of unusually high RNase activity. cDNAs were prepared from total RNA extracts. Actin-1, 5′-AA(C/T)TGGGA(C/T)GA(C/T)ATGGA(A/G)AA-3′, and actin-2, 5′-GCCAT(C/T)TC(C/T)TG(C/T)TC(A/G)AA(A/G)TC-3′, as well as RpL8-1, 5′-GAGTCATCCGAGCTCA(A/G)(A/C)G(A/G/C/T)AA(A/G)GG-3′, and

RpL8-2, 5′- CCAGCAGTTTCGCTT(A/G/C/T)AC(C/T)TT(A/G)TA-3′, were used to detect *actin* and *RpL8* gene expression as endogenous controls. The following GSPs were used to detect *AtraPBP1, AtraPBP2, AtraGOBP1, AtraGOBP2*, *AtraCSP*, and *AtraGSP* gene expression:

AtraPBP1-1 5′-CTCACAGGCTGTGCCATCAAGTGTCTCTC-3′; AtraPBP1-2 5′-CAACTTCCATGTTAGGAGCCCATTTGAGG-3′; AtraPBP2-1, 5′-ATCATGTGCATGGCCGCCAAGCTGGACCTG-3′; AtraPBP2-2, 5′-CCACGTCCAGGGTGCGGGCGCAGTGGTCGC-3′; AtraGOBP1-2, 5′-AAAAGTGACCGTTGCATAGAAGCTTATGCG-3′; AtraGOBP1-3, 5′-GGCGAGGTCCTGGCCTCCCAGATGGTGCAG-3′; AtraGOBP2-1, 5′-GAAGTGGTGGACCGCGAGCTGGGCTGCGCC-3′; AtraGOBP2-6, 5′-ACCCGGTCGCACTCGTCCGCGATGTCGTCG-3′; AtraCSP-1, 5′-GGGCAAGTGCACGCCGGAAGGAAAGGAAC-3′; AtraCSP-5, 5′-CACGATGCCCTTGGCCCTTGCGCGGTCTTC-3′; AtraGST-3, 5′-GATAAAGGTAGTCCTCCATTGCGAAAGGC-3′; AtraGST-4, 5′-GGACCATGTTCAGATCTAAGCCGATAGCC-3′. PCR was run at 94°C for 30 sec, with 22 cycles of annealing at 55°C for 30 sec, and extension at 72°C for 1 min. PCR product was separated on 0.8% agarose gel, and photographed and cropped using Gel Doc EQ and Quantity One (Bio-Rad). Tissues (antennae, legs, wings, thorax, abdomen) from adults of both sexes were dissected on ice under a light microscope. Total RNA was extracted using TRIzol Reagent (Invitrogen, Carlsbad, California) and first-strand cDNAs were synthesized from 0.5 µg RNA SuperScript II Reverse Transcriptase (Invitrogen) and an oligo(dT) primer, following manufacturer's instructions. Integrity of cDNA templates was confirmed by amplification of “housekeeping” genes encoding actin and RpL8. Gene specific primers used for tissue-specificity study of AtraSNMP1, AtraGST and AtraABPX are as follows. AtraSNMP1up: 5′- CAGCTCTGAAGAAGGAAAACGTCG-3′; AtraSNMP1do: 5′- TTCAGAAGTTCCGGATCGCTGTC-3′; AtraGSTup: 5′- ATGCCAGCGCAAGCTATTAAGTT-3′; AtraGSTdo: 5′- CTTTCTTCGTTCTGGCAAACCAT-3′; AtraABPXup: 5′- CAAGATGGTGCACGACAACTGCG-3′; AtraABPXdo: 5′- TCCTTCTTGTTGGCCTTCTGCCA-3′

### Phylogenetic analysis of moth PBPs

Amino-acid sequences of PBPs identified in different moth species (57 proteins from 33 species have been retrieved from GenBank database) have been combined to AtraPBP1 and AtraPBP2 to create an entry file for phylogenetic analysis in MEGA 4.0.2 [33]. An unrooted consensus neighbor joining tree [34] was calculated at default settings with pairwise gaps deletions. Branch support was assessed by bootstrap analysis based on 1000 replicates.

### Protein expression, biophysical studies and binding assays

pBluescript clones including 3′- and 5′-regions of AtraPBP1 cDNA were used as template for PCR. Following GSPs including recognition sites of *Kpn* I and *Xho* I were used: AtraPBP1-5, 5′-CCGGGGTACCCTCGCCGGAGATCATGAAGG-3′; AtraPBP1–4, 5′-CCGCTCGAGTTAGACTTCAGCCAGGACCTC-3′. *PfuUltra* High-Fidelity DNA Polymerase (Stratagene) was selected as DNA polymerase. PCR: 95°C for 2 min; 30 cycles of 95°C for 30 sec, 40°C for 30 sec and 72°C for 1 min; 72°C for 10 min. After gel-purification 500 bp of PCR product was inserted into *Eco* RV recognition site of pBluescript SK (+) (Stratagene). DNA sequences of two clones were confirmed. Sixteen micrograms of DNA mixed with both clones was treated with 40 U of *Kpn* I (New England Biolabs, Ipswich, MA) at 37°C for 3 h and subsequently re-purified by QIAquick PCR Purification Kit (Qiagen, Valencia, CA). Purified DNA was treated with T4 DNA polymerase (New England Biolabs) at 12°C for 20 min to remove 5′-protruding single strand DNA fragment. Reaction of T4 DNA polymerase was stopped at 75°C for 10 min. After re-purification of DNA by QIAquick PCR Purification Kit, DNA was digested with 40 U of *Xho* I at 37°C for 3 h. 500 bp of DNA fragment was gel-purified and ligated into previously digested pET-22b(+). Digestion of 1.2 µg of pET-22b (+) plasmid DNA (Novagen, Gibbstown, NJ) was done with 6 U of *Msc* I and 5 U of *Xho* I (New England Biolabs) at 37°C for 3 h. After heat denaturing enzymes at 65°C for 20 min, digested plasmid DNA was gel-purified. Connection between pET vector and AtraPBP1 DNA insert was confirmed by sequencing using T4 terminator primer (Novagen).

Expression of non-labeled AtraPBP1 was performed in LB medium with transformed BL21(DE3) cells [3]. Proteins in the periplasmic fraction were extracted with 10 mM Tris-HCl, pH 8 by using three cycles of freeze-and-thaw and centrifuging at 16,000 ×*g* to remove debris [36]. The supernatant was loaded on a Hiprep™ DEAE 16/10 column (GE Healthcare, Piscataway, NJ). Separations by ion-exchange chromatography were done with a linear gradient of 0–500 mM NaCl in 10 mM Tris-HCl, pH 8. Fractions containing the target protein were further purified on a Q-Sepharose Hiprep™ 16/10 column (GE Healthcare) and, subsequently, on a Mono-Q HR 10/10 column (GE Healthcare). PBP fractions were concentrated by using Centriprep-10 (Millipore, Billerica, MA) and loaded on a Superdex-75 26/60 gel-filtration column (GE Healthcare) pre-equilibrated with 150 mM NaCl and 20 mM Tris·HCl, pH 8. Highly purified protein fractions were concentrated by Centricon-10, desalted on four 5-ml HiTrap desalting columns (GE Healthcare) in tandem and by using water as mobile phase, analyzed by LC-ESI/MS, lyophilized, and stored at −80°C until use. The concentrations of the recombinant proteins were measured by UV absorbance at 280 nm in 20 mM sodium phosphate, pH 6.5 and 6 M guanidine HCl by using the theoretical extinction coefficients calculated with EXPASY software (http://us.expasy.org/tools/protparam.html). LC-ESI-MS was performed with a LCMS-2010 (Shimadzu, Kyoto, Japan). HPLC separations were done on a ZorbaxCB C8 column (150×2.1 mm; 5 µm; Agilent Technologies, Palo Alto, CA) with a gradient of water and acetonitrile plus 2% acetic acid as a modifier. The detector was operated with the nebulizer gas flow at 1.0 l/min and the curved desolvation line and heat block at 250°C. ^15^N-labeled AtraPBP1 [43] was prepared as previously described for BmorPBP1 [4]. CD spectra were recorded by using a J-810 spectropolarimeter (Jasco, Easton, MD) with 0.2 mg/ml AtraPBP1 in either 20 mM ammonium acetate, pH 7 or 20 mM sodium acetate, pH 5. Fluorescence spectra were recorded on a Shimadzu RF-5301 PC spectrofluorometer with 10 µg/ml of AtraPBP1 in 20 mM of one of the following buffers: sodium acetate, pH 4, 5 and ammonium acetate, pH 5.5–7. The protein solution was excited at 280 nm and the emission spectra were recorded between 285 and 420 nm. Excitation and emission slits were set at 1.5 and 10 nm, respectively. NMR was obtained on a Bruker Avance 600 MHz spectrometer equipped with a four-channel interface and triple-resonance cryogenic probe. The 15N–1H HSQC spectra were obtained with ^15^N-labeled 0.5 mM AtraPBP1 in 95% H_2_0 and 5% ^2^H_2_O, with pH adjusted to 4.5 (20 mM sodium acetate), 5.5 (20 mM sodium acetate), and 7.4 (20 mM sodium phosphate).

Binding was measured by incubating AtraPBP1 with test ligands, separating unbound and bound protein, extracting pheromone from the latter sample, and analyzing by gas chromatography, according to a previously reported protocol [5]. After lowering pH to release ligand, bound protein fractions were extracted and analyzed by gas chromatography (GC) for quantification and by GC-mass spectrometry (GC-MS) for confirmation of ligand identity. GC and GC-MS were done on a 6890 series GC and a 5973 Network Mass Selective Detector (Agilent Technologies, Palo Alto, CA), respectively. Both instruments were equipped with the same type of capillary column (HP-5MS, 25 m×0.25 mm; 0.25 µm; Agilent Technologies) operated under the same temperature program (150°C for 1 min, increased to 250°C at a rate of 10°C/min, and held at the final temperature for 10 min). Pure pheromone samples, including isomers of 11,13-hexadecadienal, were supplied by Bedoukian Research Inc. Each compound was tested at least five times. Test compounds were incubated with AtraPBP1 in the ratio of 10∶1, ligand∶protein. For competitive binding assays, all ligands were added to a protein solution at the same concentration.
